# Orden de no reanimar y limitación del esfuerzo terapéutico: desafíos éticos en equipos sanitarios en Chile

**DOI:** 10.18294/sc.2024.4821

**Published:** 2024-06-05

**Authors:** Ana López-Ávila, Edith Rivas-Riveros, Maggie Campillay-Campillay

**Affiliations:** 1 Magíster en Epidemiología Clínica. Enfermera clínica, Hospital Regional de Talca, Chile. a.lopez89117@gmail.com Hospital Regional de Talca Enfermera clínica Hospital Regional de Talca Chile a.lopez89117@gmail.com; 2 Doctora en Enfermería. Directora, Maestría en Enfermería, Universidad de La Frontera, Temuco, Chile. edith.rivas@ufrontera.cl Universidad de La Frontera Universidad de La Frontera Temuco Chile edith.rivas@ufrontera.cl; 3 Doctora en Enfermería. Coordinadora, Maestría de Investigación en Metodologías Cualitativas para la Salud, Universidad de Atacama, Copiapó, Chile maggie.campillay@uda.cl Universidad de Atacama Universidad de Atacama Copiapó Chile maggie.campillay@uda.cl

**Keywords:** Orden de no Resucitar, Cuidado en el Final de la Vida, Bioética, Personal de Salud, Chile

## Abstract

El propósito de este trabajo es profundizar en los aspectos éticos que experimenta el equipo de salud cuando reciben la indicación de limitar el esfuerzo terapéutico o la orden de no reanimar. Desde un paradigma interpretativo, cualitativo y con un enfoque de análisis de contenido, se realizó un proceso basado en tres fases: preanálisis en el que se identificaron las categorías, la proyección del análisis y el análisis inductivo. Durante 2023, se realizaron entrevistas en el entorno clínico de un hospital de alta complejidad en Chile a 56 miembros de equipos de salud de unidades críticas y urgencias, de las que emergieron cuatro categorías: a) riesgo de vulnerar los derechos de los pacientes al utilizar la orden de no reanimar, y limitación del esfuerzo terapéutico; b) brecha en la interpretación del marco legal que aborda la atención y cuidado de pacientes al final de la vida, o con enfermedades terminales por parte del equipo de salud; c) conflictos éticos de la atención al final de la vida; y d) el cuidado eficiente o el cuidado holístico en pacientes con enfermedad terminal. Existen brechas importantes en la formación en bioética y aspectos del buen morir en los equipos de salud que se enfrentan a la orden de limitar el esfuerzo terapéutico y no reanimar. Se sugiere capacitar al personal, y trabajar una guía de consenso para abordar los aspectos éticos del buen morir.

## INTRODUCCIÓN

Los diferentes acontecimientos históricos y sociales que cuestionan el paternalismo asistencial, los riesgos y beneficios de las nuevas tecnologías de la salud han contribuido al desarrollo de la bioética asistencial; cuestionando con ello la fe ciega en la medicina, para dar paso hacia una sociedad más reflexiva en que se demanda mayor responsabilidad de quienes la ejercen^1^. Uno de los casos más discutidos sobre derechos de los pacientes, y la limitación del esfuerzo terapéutico fue la solicitud de los padres de Karen Ann Quinlan que, en el año 1976 en EEUU, se opusieron a mantener el respirador para prolongar artificialmente la vida de su hija en estado vegetativo, generando un conflicto ético y legal con el equipo médico responsable a cargo de su tratamiento. Estos eran partidarios de mantener el soporte respiratorio, a como diera lugar, ya que su retiro fue considerado por ellos como moralmente cuestionable^2^. El juez sentenció en favor del retiro del respirador y Karen sobrevivió con signos de deterioro físico por un tiempo más, y con ayuda de alimentación e hidratación enteral. Como respuesta a este caso, se comienzan a instalar los comités de ética asistencial a nivel hospitalario, de forma de contribuir a la deliberación ética, asesorar a los equipos en conflictos éticos de la práctica clínica, y evitar el encarnizamiento terapéutico^2^^,^^3^.

Junto con el avance sostenido del uso de la tecnología en la medicina y el aumento de las expectativas de vida de la población, al mismo tiempo emergen las discusiones sobre el derecho a la muerte digna, con importantes matices socioculturales. Principalmente porque las decisiones al final de la vida responden a patrones de filiación familiar, religiosos, y concepciones de la muerte que varían entre las distintas regiones del mundo^4^. Ejemplo de esto en el mundo occidental, es la conducta evitativa de la población para hablar de la muerte, lo que ha dificultado la preparación de los profesionales de la salud para enfrentar adecuadamente la etapa de duelo anticipado con el paciente hospitalizado, con enfermedad grave, y la relación del moribundo con su familia. En este sentido, morir no es solo una experiencia del equipo de salud y el paciente, sino una responsabilidad compartida con la institución, familiares, amigos, religiosos y grupos comunitarios. Los estudios de seguimiento a la atención y cuidado de pacientes moribundos realizados por Kübler Ross describieron que el equipo responsable del cuidado de estos pacientes se preocupaba principalmente del cuidado físico y médico. Sin embargo, las personas moribundas pasaban largo tiempo solas, ya que el equipo evitaba el acompañamiento psicosocial y espiritual, al no tener resuelta su propia perspectiva de la muerte. Por ello, se vuelve fundamental la capacitación permanente y la formación humanizada de los equipos de unidades críticas^5^.

En Europa y posteriormente en América Latina, los criterios de la Joint Commission on Accreditation of Health Care Organizations fueron incorporados a la acreditación de la calidad de los hospitales, reforzando la institucionalización de los debates éticos a través de los comités de ética que han permitido avanzar en temas de la dignidad, autonomía y final de la vida de los pacientes que ingresan a las unidades críticas de los hospitales más complejos^3^.

En Chile, la prolongación del proceso de morir ha estado permeada por la limitación del esfuerzo terapéutico y la orden de no reanimar, concepciones que se abordan en la Ley 21375 y la Ley 20584. Al respecto, estas disposiciones modificaron la forma de relacionarse entre los pacientes y el equipo de salud, en favor del derecho de las personas para ser tratadas con mayor dignidad durante su trayectoria terapéutica, acceder a un cuidado integral, y alivio frente a padecimientos asociados a una enfermedad terminal o grave^6^^,^^7^.

El concepto de limitación del esfuerzo terapéutico se ha ido modificando en el tiempo, ya que no se limita solo al esfuerzo terapéutico, sino que incluye un ajuste a los objetivos terapéuticos, por lo que algunos autores han optado por utilizar el concepto de adecuación del esfuerzo terapéutico^8^. Conceptualmente, la limitación del esfuerzo terapéutico corresponde al *retiro* o *no inicio* de medidas terapéuticas, porque resultan inútiles o fútiles sobre la base de la situación médica del paciente. En este sentido, seguir con medidas terapéuticas solo conseguiría prolongar la vida biológica, pero sin posibilidad de proporcionar una recuperación funcional o una mínima calidad de vida^9^. Además, en fases terminales de la enfermedad, es materia de discusión cuándo el enfoque del tratamiento ha dejado de ser curativo y, por lo tanto, el manejo se orienta a aliviar el dolor, el sufrimiento, y obtener el máximo confort del paciente, sin prolongar el proceso de morir, respetando los deseos del paciente y la familia, y de forma razonablemente consistente con los estándares éticos, culturales y clínicos de cada país^8^^,^^10^. Por tanto, la limitación del esfuerzo terapéutico busca una adecuación proporcional del tratamiento, como consecuencia de una deliberación entre paciente y profesional, fundamentada en una relación participativa y de confianza como manifestación de respeto por la dignidad personal y de humanidad del paciente^10^ y, en un sentido práctico, se considera una ayuda para que el equipo de salud disponga de un marco de actuación o de manejo durante el proceso asistencial^11^.

Desde el punto de vista de los fundamentos bioéticos que sustentan la limitación del esfuerzo terapéutico están: cumplir con los fines de la medicina, evitar el daño y el sufrimiento innecesario, permitir una muerte tranquila, respetar los valores y deseos del paciente, mantener la proporcionalidad de las terapias, y buscar la racionalidad en el uso de los recursos basado en el principio de justicia^10^. Así, la proporcionalidad del esfuerzo terapéutico se refiere siempre a una situación clínica particular, que debe ser producto de una amplia deliberación del equipo médico, y siempre dejando abierta la posibilidad de una reconsideración^12^.

Una de las implicancias de la limitación del esfuerzo terapéutico es la restricción de ingreso de un paciente a una unidad de cuidados intensivos. Se han intentado definir criterios para limitar la admisión de pacientes que no se van a beneficiar de esta decisión, considerando la necesidad de monitorización activa, la reversibilidad del proceso y la imposibilidad de que el tratamiento necesario se realice fuera de esta unidad^13^. Así, la toma de decisiones en este sentido, responde a las condiciones del paciente no solo físicas, sino de conciencia para participar, en lo posible, de estas decisiones, razón por la cual los criterios a considerar deben ser técnicos y éticos. Estas decisiones no están exentas de conflictos morales, en especial, porque deben tener en consideración que lo más probable es que las decisiones sean por subrogación de los miembros de la familia de pacientes en riesgo vital^14^. Los criterios técnicos se relacionan con la probabilidad de supervivencia, y los beneficios al utilizar tecnología de avanzada para mantener a los pacientes con menos sufrimiento y mejor calidad de vida. En este sentido, la experiencia internacional relacionó el buen morir^15^ con el uso de la orden de limitación del esfuerzo terapéutico dentro de las primera 48 horas de ingresado el paciente. Por ello, el proceso debe quedar claramente registrado y documentado en la ficha clínica. Esto incluye justificar la indicación y dejar constancia si la familia está o no en conocimiento de tal decisión, y si la comparte o no. Además, existe la obligación médica y el deber moral de no abandonar al paciente durante el proceso de muerte, garantizar las medidas necesarias de bienestar, cuidados, analgesia y sedación, asegurando la ausencia de dolor físico, o psíquico^15^.

Por su parte, la orden de no reanimar es la instrucción por adelantado, en la que un médico indica al equipo de salud que no deben iniciar reanimación cardiopulmonar en un paciente si se detiene su corazón, o si deja de respirar^16^,^17^.

Es un tema relacionado con la dignidad humana, los derechos del paciente, el proceso de morir, y que requiere de habilidades de comunicación interpersonal con el paciente, equipo y familia. La limitación del esfuerzo terapéutico está justificada desde una perspectiva de justicia (evitando la futilidad), no maleficencia (evitando el encarnizamiento terapéutico), beneficencia (respetando la dignidad de la persona) y, de autonomía del enfermo (respetando su voluntad o la de su familia)^18^. En la decisión de no reanimar adquieren especial preeminencia los principios éticos de proporcionalidad terapéutica, el ejercicio de la libertad responsable, y la inviolabilidad de la vida humana, en la medida que tal determinación trae como consecuencia cierta, ineludible e inmediata muerte de la persona afectada por un paro cardiorrespiratorio^12^. En este escenario, la voluntad anticipada del paciente debe ser respetada, aunque no siempre es posible que todos los pacientes quieran hablar de esta posibilidad, y lo mismo puede ocurrir con la familia. Asimismo, los médicos consideran que hablar este tema con el paciente y la familia es desafiante, ya que puede alterar la relación médico-paciente y restar esperanza a la familia^16^. Otro aspecto sensible es la privacidad con que se maneje este tema, considerando que los códigos que se adopten para comunicar la orden de limitar el esfuerzo terapéutico al personal sanitario no deben ser descifrables para los otros pacientes. Al respecto, la educación del personal de salud se vuelve esencial para enfrentar esta situación, respetando la confidencialidad y dignidad del proceso de morir de todo paciente^19^.

Por ello, el objetivo de este trabajo es profundizar, desde un enfoque cualitativo, en los aspectos bioéticos que experimenta el equipo de salud cuando recibe la indicación de limitar el esfuerzo terapéutico y la orden de no reanimar. Se espera con ello, aportar a la discusión informada de los equipos sanitarios, en el cuidado centrado en el paciente, y como sujeto de derecho.

## MÉTODO

Se realizó un estudio exploratorio desde un paradigma interpretativo y enfoque cualitativo. La muestra fue intencional, conformada por los profesionales y técnicos de la salud (médicos, enfermeras, kinesiólogos, y técnicos de enfermería) que se desempeñaban en las unidades de paciente crítico, unidad de hospitalizados de medicina interna, y servicio de urgencias de un hospital de alta complejidad de la zona sur de Chile. Estas unidades se caracterizan por proporcionar atención a pacientes que ingresan en riesgo vital, por lo que es habitual que, en las tareas de estos equipos, se utilice tecnología de monitoreo cardiaco, asistencia respiratoria, catéteres, y otras tecnologías médicas de avanzada, y se enfrenten a decisiones de final de vida. Se consultó a la dirección del establecimiento por el número de funcionarios que trabajan en esas unidades, y se les hizo llegar a los jefes de servicios una invitación voluntaria para que pudieran participar al menos 20 personas por servicio. Estos hicieron el nexo con sus equipos, recibiendo la respuesta positiva de 20 informantes por cada unidad. Sin embargo, en la unidad de urgencia, cuatro de los funcionarios voluntarios posteriormente desistieron de participar, completando una muestra de 56 sujetos en total. El periodo de entrevistas se extendió por seis meses durante el año 2023.

Los criterios de inclusión consideraron a miembros de equipos que realizaran actividades de atención directa a los pacientes, y con al menos un año de experiencia en alguno de los servicios de interés. La técnica de recolección de datos fue a través de entrevistas semiestructuradas, y se utilizó un guion con cuatro preguntas orientadoras, entre las que se incluyó: “¿qué entiende por limitación del esfuerzo terapéutico, orden de no reanimar y su relación con los cuidados al final de la vida y el buen morir de los pacientes?”. Esto permitió orientar el curso de la entrevista con el fin de profundizar en los objetivos del estudio, y dar mayor énfasis a las temáticas emergentes. La recolección de los datos se inició una vez obtenida la evaluación y aprobación del Comité de Ética Científico de la Universidad de La Frontera consignada en el acta No. 67/23, junto con la autorización por escrito de la dirección del establecimiento donde se desarrolló la investigación.

Las entrevistas se realizaron en una sala del mismo hospital, y se respetaron los protocolos de prevención de covid-19. El promedio de duración de las entrevistas fue de 67 minutos, y el rango de tiempo de duración fue entre 45 a 90 minutos. Las entrevistas fueron consentidas con registro físico, y se utilizó un reproductor digital para la grabación de voz. Además, se tomaron notas de campo para registrar cualquier observación que sirviera para la posterior interpretación de los datos. La transcripción se realizó de forma fidedigna, se creó un archivo de almacenamiento con anonimización de las entrevistas y asignación de un código de informante “ID” acompañado de un número correlativo. Finalmente, se optó por análisis manual, al no contar con acceso a un software con licencia vigente.

El criterio de credibilidad consideró la triangulación, al realizar un cruce de datos entre la investigadora principal y las investigadoras secundarias, quienes contribuyeron a la interpretación, experiencia en estudios cualitativos y retroalimentación, para una mayor reflexividad basada en el compromiso e interés por la temática. La saturación se alcanzó, ya que una vez establecidas las categorías emergentes fue posible identificar que existían similitudes, y escasa información nueva entre los informantes. De igual forma, siendo los resultados de un solo hospital, pero las características similares en la formación de los profesionales sanitarios en nuestro país, posiblemente se puedan obtener resultados transferibles a otros equipos de unidades críticas de otros hospitales de Chile^20^.

Se trata de un análisis de contenido cualitativo, siguiendo la vertiente cualitativa de Bardin^21^. Se realizó: a) un preanálisis en el que se identificaron las categorías, b) una segunda fase de proyección del análisis, y c) una tercera fase, en que se analizaron los datos de forma lógica e inductiva, para cumplir con los objetivos del estudio. El preanálisis fue realizado de manera independiente entre la investigadora principal y las colaboradoras. Se listaron todas las posibles categorías que progresivamente aparecieron como resultado de las distintas rondas de lectura que se realizaron de los datos. En la segunda fase, se identificaron las categorías temáticas consideradas sustantivas, tanto por la tendencia del contenido, como la importancia asociada a los objetivos. Finalmente, en la tercera fase, las investigadoras optaron por priorizar ciertas categorías, ya que muchas eran propiedades o características de otras. En suma, las categorías temáticas emergentes fueron: a) riesgo de vulnerar los derechos de los pacientes al utilizar la “orden de no reanimar” y la “limitación del esfuerzo terapéutico”; b) brecha en la interpretación del marco legal; c) conflictos éticos de la atención al final de la vida; y d) el cuidado eficiente o el cuidado holístico en pacientes con enfermedad terminal.

## RESULTADOS Y ANÁLISIS

### Caracterización de la muestra

Se entrevistaron a 56 profesionales y técnicos de salud (médicos, enfermeras, kinesiólogos, y técnicos de enfermería), de los servicios de paciente crítico, medicina y urgencias, con variados años de experiencia, y con un promedio de edad de 37 años (Tabla 1).

**Tabla 1:** Caracterización de la muestra. Talca, Chile, 2023.

Características de los/las informantes	No. de informantes	Promedio de edad	Unidad			Promedio de años de experiencia
			Unidad de paciente crítico	Medicina	Urgencia	
Técnicos	20	38	6	9	5	10,5
Enfermeras	18	35	7	5	6	9,0
Kinesiólogos(as)	8	34	4	2	2	9,8
Médicos(as)	10	41	3	4	3	14,6
Totales	56	-	20	20	16	-

Fuente: Elaboración propia.

### Riesgo de vulnerar los derechos de los pacientes al utilizar la orden de no reanimar, y la limitación del esfuerzo terapéutico

Los informantes mencionan que existe un uso habitual de estas órdenes en las unidades estudiadas, sin embargo, se observan variaciones en el equipo al interpretarlas y ejecutarlas en la práctica clínica. En este sentido, la mayoría menciona que la *orden de no reanimar* es una orden literal, es decir, el paciente con esta indicación no debe recibir medidas de reanimación cardiopulmonar por su avanzado estado de compromiso general.

“*En el caso de que el paciente haga un paro cardiorrespiratorio no se le va a hacer reanimación cardiopulmonar, no se va a intubar si lo requiere, o algo así. No se reanima. Eso no quiere decir que no se le siga pasando su tratamiento como corresponde, su alimentación y todo, hasta el último suspiro*”. (ID 34)

En cambio, al enfrentarse a la indicación de *limitación del esfuerzo terapéutico,* mencionan que si bien comprenden el contexto médico que lo justifica, existen dificultades para decidir cómo actuar adecuadamente frente al paciente:

“*Yo pensaba que era lo mismo, cuando lo limitan y al final no se reanima. Cuál es la diferencia, no sabría decirlo*”. (ID 4)

“C*uando nos indican limitación del esfuerzo terapéutico es más que nada limitar las terapias o procedimientos invasivos, cuando el pronóstico es malo en el paciente, y en realidad llega como a un techo. Por ejemplo, puede haber medidas de soporte, pero ya no vamos a innovar en otras cosas más. Entonces hay una limitación en cuanto a la actividad. Bueno, a nosotros igual acá de repente se nos genera un poco de problema, yo creo interpretativo. De repente, la persona que lo indica también, ¿qué es lo que queremos en el fondo?, ¿queremos mantener al paciente, queremos ya quitarle algunas medidas de soporte?, y ahí yo creo que hay problemas de interpretación, porque a veces, pasa un turno que lo limita, y de repente el otro médico indica otras cosas, entonces falta a lo mejor ponerse de acuerdo con esas cosas*”. (ID 37)

A nivel global, la literatura menciona que la aplicación de dichos términos genera complicaciones prácticas y éticas, porque existe una gran cantidad de expresiones semánticas alternativas^22^. Por otra parte, habitualmente estas órdenes se asocian a las siglas ONR (orden de no reanimar), o LET (limitación del esfuerzo terapéutico) de uso extendido junto a otras siglas entre el personal de salud, y que pueden provocar interpretaciones erróneas, lo que implica siempre la posibilidad de error en la ejecución de las indicaciones^23^, especialmente, entre el personal con menos experiencia. Otro aspecto relevante, es que las decisiones terapéuticas relacionadas con el pronóstico y el manejo del paciente son de responsabilidad médica y, en el caso de las enfermeras u otros profesionales sanitarios que participan en la ejecución de la orden de no reanimar y limitación del esfuerzo terapéutico, no siempre participan de la deliberación de los aspectos que sean más aconsejables y beneficiosos para el paciente, lo que puede incidir en problemas de salud mental al ejecutar la orden. Aunque cada vez existe mayor tendencia a consultar a todo el equipo, lo que sin duda contribuye a que todo el equipo pueda afrontar de mejor forma estas indicaciones^19^^,^^24^.

En este sentido, el principal obstáculo para los equipos sanitarios es hacer una correcta interpretación de las indicaciones, lo que puede influir en las acciones para asegurar que los pacientes mueran con dignidad y comodidad. Más allá de la estandarización de las siglas, los conocimientos sobre los cuidados al final de la vida, y familiarizarse con el marco legal de cada país es fundamental para disminuir el riesgo de error, y prevenir la vulneración de derechos de los pacientes^19^. De acuerdo a la American Nurses Association^25^ apoyar las decisiones del paciente al final de la vida, debe darse de manera coherente con sus preferencias y valores, y se relaciona con una comprensión adecuada de la terminología médica utilizada. Por lo que se debe desalentar usar acrónimos, siglas y abreviaturas con el paciente y la familia, ya que habitualmente provocan problemas de comunicación.

Al consultar sobre los beneficios que tiene, para los pacientes al final de la vida, la limitación del esfuerzo terapéutico, las personas entrevistadas lo asocian a una mejor calidad de vida, confort, muerte digna y no abandono del paciente. Estas ideas fundamentan los beneficios que tiene para los pacientes el uso de la limitación del esfuerzo terapéutico de manera de no prolongar el sufrimiento físico persistente, que enfermedades graves y terminales pueden provocar a estas personas^6^. Además, si bien el uso de estas indicaciones en pacientes críticos provoca, en los equipos profesionales, angustia moral y fuertes emociones que son difíciles de manejar^26^, participar en la planificación de los cuidados, junto con mejorar la condición de fin de la vida de estos pacientes, genera tranquilidad en los equipos clínicos bajo la idea de que han actuado haciendo todo lo posible por el paciente:

“*Si el paciente está consciente, tiene miedo de sentir dolor, tiene miedo de sentirse ahogado. Entonces, para mí, en cuanto a síntomas, es que no tenga que sentir eso. Y en cuanto a la parte emocional, es también estar acompañado, no pasar este proceso solo. También, que el paciente, si tiene deseo de ver a personas que no ha visto en mucho tiempo, cumplir con esos últimos deseos que son cumplibles, y eso es tener un buen morir para mí*”. (ID 16)

“*Si uno hace lo máximo que pudo para que tuviera un buen morir, uno se va más tranquilo. Eso es una enseñanza, porque a veces, por cosas equis, no se logra. Siempre uno cuando pasa después piensa, ‘pude haber hecho esto, pude haber hecho esto otro’, te da como más confianza para cuando existe un plan, eso te da una experiencia en el buen morir de los pacientes*”. (ID 6)

Al consultar por cuidados al final de la vida, los informantes lo asocian con atención y acompañamiento holístico, relevando en esta etapa la importancia del cuidado en todas las dimensiones humanas, física, psicológica, y social:

“*Cuidados al final de vida es darle confort al paciente. Sobre todo, en enfermedades terminales, manejo del dolor, acompañamiento familiar, atención psiquiátrica, psicológica, sobre todo a los familiares*”. (ID 9)

Con relación a los *cuidados paliativos*, esto se asocia a proveer cuidados en pacientes terminales o con cáncer. En este punto, se menciona especialmente el manejo del dolor, el confort y el acompañamiento, muy similar a los cuidados al final de la vida, sin embargo, los informantes identifican a los pacientes que, por su enfermedad, están viviendo una agonía más dolorosa:

“*Los cuidados paliativos son, por ejemplo, los pacientes oncológicos que ya como que están muy complicados de su enfermedad, de su patología. Es como brindarles los medicamentos que sean necesarios para que no tengan mucho dolor, darle sus medicamentos, como los cuidados de buen morir, eso entiendo yo como cuidados paliativos. como que el paciente llegue a su último día de una forma, sin dolor, y tranquilo*”. (ID 4)

El cuidado de pacientes con enfermedades terminales dolorosas, como el cáncer, deterioran la calidad de vida del paciente y afectan su entorno familiar. El dolor, en estos casos, no se refiere solo a la percepción física, sino un sufrimiento asociado también a la angustia psicológica, pérdida de las actividades sociales y el duelo familiar. El estudio realizado por Parra *et al*.^27^ en un servicio de pediatría oncológica describió el importante rol que cumple la familia para el control del dolor al ser un proceso multifactorial, ya que junto con el acompañamiento permanente de la familia a niños y niñas, se requiere que estos autoricen el tratamiento analgésico para evitar el sufrimiento físico por su condición heterónoma. Otros estudios han descrito la importancia del elemento personal, ya que cada ser humano vive su proceso de dolor de forma distinta. En este punto, los informantes utilizan el concepto de *“confort del paciente”* para referirse a toda actividad que favorezca la comodidad, control del dolor y acompañamiento significativo del paciente, especialmente, de sus familias. La revisión de Zamán *et al*.^28^ sobre el buen morir es coincidente con los hallazgos de este estudio, al enfatizar que el paciente terminal debe morir en el lugar que prefiera, con alivio del dolor y sin angustia psicológica, con apoyo emocional de sus seres queridos y toma de decisiones autónoma, cuando sea posible, y evitar prolongar la vida limitando las intervenciones inútiles.

El dolor es una de las experiencias humanas más misteriosas que existen, el sufrimiento físico de pacientes terminales se asocia también al sufrimiento psicológico y espiritual, lo que se vive de manera personal y cuyo sentido es materia de debate permanente en la sociedad. Al respecto, algunos grupos encuentran en el sufrimiento un sentido espiritual, que fortalece la dignidad humana; mientras, para otros, sufrir es un sin sentido que resta dignidad a la vida humana^29^. Lo que está claro es que el sufrimiento se asocia a la condición de seres sintientes, expone nuestra condición de vulnerabilidad y altera el bienestar de la persona que lo padece^30^. Basado en esto, el equipo de salud debe conocer ¿cuál es el significado y sentido que el paciente terminal le ha dado a su propio dolor?, ¿hasta dónde está dispuesto a tolerar?, y ¿en qué momento requerirá apoyo medicamentoso para sobrellevarlo?

Para las personas entrevistadas, tanto los cuidados al final de la vida, como los cuidados paliativos incluyen mantener al paciente terminal aliviado del dolor y sufrimiento. Esto es coincidente con la literatura que describe la importancia de los equipos sanitarios que abogan por una muerte digna, consideran aliviar el dolor y dar condiciones de confort adecuadas. Por otra parte, se requiere de buena comunicación -especialmente con la familia, amigos y otras figuras significativas- para dar alivio psicológico y espiritual en el momento en que lo requiera el paciente^31^.

### Brecha en la interpretación del marco legal

Las personas entrevistadas mencionan que el marco legal en Chile, que regula la atención al final de la vida y enfermedades terminales, es insuficiente para contar con adecuados protocolos de atención, mientras que otros miembros del equipo desconocen el marco legal que regula estos casos. Además, se mencionan aspectos coercitivos de la ley, al no prestar atención adecuada a este grupo de pacientes.

En este sentido, existen brechas de conocimientos sobre los aspectos legales que regulan estos temas, aspecto que requiere de abordaje urgente, ya que limita el accionar profesional, y pone en riesgo el ejercicio de los derechos de los pacientes:

“*Yo creo que, primero, esto de la limitación del esfuerzo terapéutico y la orden de no reanimación pasa por una frontera entre lo médico y lo legal, porque cuando uno ve estrictamente cómo se implementa la limitación del esfuerzo terapéutico, cada país debe tener una legislación al respecto y Chile no la tiene. Entonces todo queda a criterio del equipo de trabajo que se encuentre cuidando al enfermo. Entonces creo que tendríamos que unificar criterios, hacer un protocolo de índole ministerial que se aplicará para tener una uniformidad nacional de criterios y que no hubiera duda en cuanto a esto*”. (ID 53)

Esta brecha de conocimientos sobre el marco legal que regula el cuidado al final de la vida en el equipo de salud es compleja, en la medida en que el marco normativo sanitario existente es el resultado de un debate social y democrático en que los profesionales de la salud deben participar y estar informados, ya que cumplen acciones fundamentales como prestadores, y responsables del cuidado de los pacientes^32^^,^^33^. Por otra parte, estas leyes se operacionalizan a través de orientaciones técnicas, que guían las decisiones y acciones que toma el equipo, como por ejemplo; la Ley 21375 que consagra los cuidados paliativos y los derechos de las personas que padecen enfermedades terminales o graves, y que dio forma a la Orientación Técnica de Cuidados Paliativos Universales^34^. Estas orientaciones se deben ir incorporando a la *lex artis,* que corresponde al conjunto de las mejores prácticas con base científica que deben orientar a los profesionales y a los establecimientos de salud a nivel nacional^33^.

Otros estudios realizados en Chile expusieron también brechas de conocimientos bioéticos, como el de Reyes *et al*.^35^ en que se encontró que el 76,6% de los médicos especialistas de un hospital tenían un déficit de conocimiento con relación al consentimiento informado de sus pacientes, vulnerando el derecho a la autonomía. Y el estudio de Strickler *et al*.^36^ sobre conocimientos bioéticos en unidades de emergencia describe dificultades para aplicar conocimientos bioéticos en la práctica clínica en médicas/os y enfermeras/os.

De acuerdo a Caro^37^, “asumir los cuidados al final de la vida como política pública sustentada en los principios bioéticos y normativa vigente debe ser: apropiada, disponible, accesible, educativa e implementada en el sistema de salud”^37^. Esto genera enormes desafíos para que los tomadores de decisiones y responsables de la administración sanitaria puedan avanzar en formación continua de los equipos sanitarios en temas de ética y aspectos legales asociados al cuidado de la salud. En Chile, ya existe un importante marco normativo que recoge los cuidados al final de la vida y que, además, está en la canasta de prestaciones de las Garantías Explícitas en Salud (GES). Este grupo de normas se inicia en el año 2012 cuando se aprueba la Ley 20584 que regula los derechos y deberes de las personas con relación a las acciones vinculadas a la atención en salud. A nivel nacional, esto marca un avance importante en la relación terapéutica entre el médico y paciente, al introducir el derecho a la autonomía en las decisiones relacionadas con su proceso salud-enfermedad, reemplazando el principio de beneficencia que determinó por mucho tiempo la relación terapéutica paternalista^33^. Posteriormente, se dicta la Ley 21375 que consagra los cuidados paliativos y los derechos de las personas que padecen enfermedades terminales o graves, con el fin de dar mayor dignidad a la trayectoria de padecimiento de una enfermedad crítica y/o terminal en las personas^6^.

### Conflictos éticos de la atención al final de la vida

Los principales conflictos éticos identificados en el estudio refieren a la edad del paciente y el esfuerzo terapéutico para mantener la vida: ser persona mayor es considerado como un criterio que influye en la decisión de limitar el esfuerzo terapéutico.

#### La edad del paciente y el esfuerzo terapéutico por mantener la vida

Las personas entrevistadas dan cuenta que la edad es un factor que está presente en la reflexión que hace el equipo de salud cuando se debate sobre limitar el esfuerzo terapéutico en algún paciente. La literatura al respecto menciona que en las unidades de cuidados intensivos, la edad y la calidad de vida que se le puede ofrecer al paciente tras un período crítico, junto con sus probabilidades de sobrevivencia, son elementos relevantes para decidir limitar el esfuerzo terapéutico. Esto genera discusión, ya que estudios realizados en unidades de cuidados intensivos a nivel global mencionan que la edad es un factor independiente del mal pronóstico^38^. Mantener o retirar las intervenciones de cuidado al final de la vida depende, principalmente, de la condición médica del paciente y de la alta probabilidad de muerte, falta de respuesta a la terapia, gravedad y mal resultado en términos de calidad de vida, y no de la edad^39^. La revisión de la literatura realizada por Araújo *et al*.^40^ describe que las personas mayores reciben en su mayoría un trato injusto, al restringírseles el acceso a exámenes diagnósticos y tratamientos por parte del equipo de salud, lo cual está asociado solo a su edad avanzada. Informan, además, que reciben trato inadecuado y violencia del equipo, quienes utilizan lenguaje inapropiado y transfieren la responsabilidad de su cuidado a sus familiares, aunque su estado mental les permita mantenerse con autonomía. Esto demuestra que el edadismo está fuertemente institucionalizado, y afectan las decisiones y oportunidades para mantener funcionalidad y mejor calidad de vida de las personas mayores. Este trato injusto es una forma de ageísmo estructural que va provocando malas condiciones de vida a las personas mayores, reafirma la discriminación por parte de la población de menor edad, y resta posibilidades para mantenerse activo hasta limitar su capacidad de autonomía^41^.

“*Hay pacientes que son adultos mayores, pacientes con múltiples comorbilidades, que quizás ya su calidad de vida se ha venido deteriorando y es más fácil afrontar que se está llegando al final de la vida por el curso natural de sus enfermedades múltiples, donde se puede afrontar un poco, ya es más esperable el desenlace*”. (ID 48)

“*Un paciente joven cuesta más limitarlo, no reanimarlo, ¿entiendes? En cambio, un paciente, no sé, porque a veces uno llega a pacientes de 100 años, es como ya, es la ley de la vida, o sea, no, no, no hagamos, ¿cómo se llama? Ay, se me olvidó el término. Cuando tú haces más de lo que deberías hacer, encarnización de la medicina*”. (ID 57)

#### Orden de limitar el esfuerzo terapéutico y el dilema de sentir que esta acción causa daño al paciente

Los informantes mencionan que, una vez retiradas las medidas de soporte vital, se vuelve fundamental mantener los cuidados básicos de confort y comodidad para el paciente. Sin embargo, dejan entrever que sienten incomodidad al ejecutar esta orden. Esto es coincidente con el estudio realizado por Velarde *et al*.^42^ en que enfermeras que ejecutaron la orden de limitar el esfuerzo terapéutico sintieron que no solo no daban tratamiento al paciente, sino que estaban contribuyendo a su fallecimiento. La relación de interdependencia que desarrollan las enfermeras y sus pacientes generan lazos afectivos profundos que pueden provocar emociones negativas frente a las acciones de final de la vida o limitar la capacidad para manejar psicológicamente la muerte de estos pacientes. De acuerdo a Sprung *et al*.^19^, las y los profesionales deben recibir formación adecuada para alcanzar cierto nivel de competencias para el cuidado de personas enfermas terminales, de manera que se vuelvan expertos en toma de decisiones, en los aspectos éticos y prácticos, y respetando la visión del paciente según su cultura y creencias.

“*Un paciente no se puede morir porque no lo estás aspirando o porque se tapó un traqueo (traqueostomía) porque no lo aspiraste. Entonces eso no es correcto. Hay que estar pendientes de los cambios de posición, pacientes que están postrados, la idea es que tampoco se te escape y agregarle un dolor más o que necesite cirugía plástica, tampoco tiene sentido seguir deteriorando al paciente y gastando recursos. En el fondo que el paciente mantenga todos los cuidados mínimos posibles y de calidad*”. (ID 41)

“*Pero hay algunos elementos éticos que son los que me tocan, por ejemplo, lo que es la permeabilidad en la vida aérea. El paciente puede estar con orden de no reanimar, pero yo no puedo permitir que la causa directa de su muerte sea que el paciente se asfixie, por ejemplo. Estaría haciendo un paliativo probablemente en ese momento*”. (ID 34)

#### El cuidado eficiente, o el cuidado holístico en pacientes con enfermedad terminal

Algunos de los informantes expresaron sentimientos de frustración por la inversión de tiempo, recursos y costos asociados al cuidado de un paciente que luego recibe orden de limitar el esfuerzo terapéutico y fallece.

“*Las personas que trabajan aquí sienten que esa inversión de tiempo puede ser perdida. Puede ser, no sé, que acompañar o estar ahí no tiene ningún valor o que va a ser una pérdida de tiempo en relación a lo que podríamos hacer con otras personas*”. (ID 32)

“*Es bien frustrante de repente que un día le vemos con todo el paciente, lo cultivemos, lo intubemos, le demos antibióticos de última línea y después a los días lleguen y le saquen todo y tú perdiste todo el trabajo que hiciste el día anterior, porque el otro médico dijo, o sea, no está limitado, no le hagamos nada y te hacen trabajar de más*”. (ID 41)

“*Exámenes no, es recurso que se pierde, nunca me lo he planteado muy profundamente, porque no es mi decisión. Pero sí le quitaría, le quitaría todo, todo*”. (ID 24)

Los estudios sobre pacientes críticos son categóricos en establecer que los costos asociados a la atención y cuidado son elevados y dependerá del tipo de tecnología médica utilizada, insumos y horas de personal dedicado al paciente^43^. Por lo que, cuestionarse el uso de recursos en un paciente que luego muere suele responder al principio de justicia, pues la finalidad es beneficiar a la mayor cantidad de pacientes. Bajo esta perspectiva, realizar acciones inútiles para sostener la vida de un paciente que tiene mal pronóstico, no solo es un recurso mal utilizado, sino es provocar encarnizamiento terapéutico y prolongar un sufrimiento innecesariamente. El dilema en este caso, es poder saber cuándo utilizar los recursos y cuándo ya no aportan bienestar al paciente, esto porque todas las vidas son importantes y requieren de un trato justo y digno^44^.

Por otra parte, la eficiencia desde la perspectiva ética es un valor instrumental ya que, de acuerdo a Gracia Guillén^45^, apela a la racionalidad económica preocupada del costo/beneficio de las acciones, característica de la civilización occidental, y que soporta su razón de ser en lo material y en lo que es útil. Por lo que sentir frustración y mencionar que “se perdieron recursos” al cuidar de una persona que luego fallece, respondería a una forma de “cuidado instrumentalizado”, probablemente inconsciente y aprendido, dado que las personas no tienen precio, sino dignidad. En este sentido, Campbell^46^ menciona que el buen morir desde una perspectiva cultural y holística, debe considerar cuatro aspectos: el lugar de la muerte, el acompañamiento, la causa de la muerte y la manera de afrontar la muerte. Por lo que la preocupación por el uso de los recursos se debe dar en el contexto de asegurar el buen morir de los pacientes.

La Ley 21375 consagra los cuidados paliativos y los derechos de las personas que padecen enfermedades terminales o graves, y en su Artículo 4 menciona “la protección de la dignidad y autonomía de las personas que padecen una enfermedad terminal o grave supone siempre respetar su vida y considerar a la muerte como parte del ciclo vital”^6^. Al respecto, la literatura sobre la actitud profesional frente a la muerte de un paciente es escasa. El estudio de Morales *et al*.^47^ describió en algunas enfermeras actitudes de indiferencia frente a la muerte de los pacientes, de manera de que no les afecte emocionalmente. Esto fue relacionado por los autores con “aceptar su propia muerte, cuidar con mucha más libertad, lo que puede traducirse en asumir responsabilidades de crecimiento personal y profesional, y permite un mayor significado de vida”^47^. Sin embargo, esta práctica de mostrar indiferencia frente a la muerte, puede evidenciar, también, dificultad para afrontar el proceso de morir, brechas en la formación, y un riesgo de transmitir mensajes erróneos a las y los estudiantes que habitualmente se ven impactados al tener las primeras experiencias de muerte^48^. Por otra parte, la muerte es una experiencia vital que requiere, según Sprung *et al*.^19^ de atención centrada en el paciente y la familia, no solo para concentrarse en aliviar los síntomas de la enfermedad, evitar o aliviar el sufrimiento físico, sino para tratar a las personas con dignidad, y abordar sus necesidades de manera holística, proporcionando los cuidados físicos, psicológicos, sociales y espirituales, de acuerdo a las preferencias del paciente o la familia.

El enfoque holístico de los cuidados considera relevante la interacción del paciente y su familia para mantener lazos afectivos y la socialización hasta el final de la vida, evitando el aislamiento social, y promueve además el acompañamiento psicológico y espiritual^49^.

#### La voluntad anticipada del paciente para limitar el esfuerzo terapéutico o ¿la decisión de otros?

En este punto, el equipo de salud valora el principio de autonomía de los pacientes, la subrogancia de la familia por sobre las decisiones tomadas por el médico:

“*Que se haga el derecho de la persona, saber que el equipo de salud no va a hacer nada si le pasa algo, quizás sea un derecho de esa persona, ¿cierto?, quizás esa persona tenga derecho a conocer su condición de no reanimar o su condición de limitación terapéutica. Quizás esa persona debiera saber*”. (ID 34)

“*Yo creo que como equipo tenemos que saber y tener la mirada de la familia, tener la mirada del paciente y no tomar nosotros la decisión por ellos*”. (ID 40)

“*La orden de no reanimación es una indicación que se realiza en base a la condición clínica del paciente, pero a veces también la define el paciente mismo y lo deja estipulado en la ficha*”. (ID 53)

“*Considero que hay que hacer partícipes a la familia también o qué es lo que quiere el paciente, porque hay pacientes que están súper vigiles, que están súper orientados en el tiempo y espacio y que están conscientes de su situación y que están en capacidad de tomar una decisión de lo que ellos quieren, pero acá los clínicos no tienen el concepto claro y por ende el hecho de orden de no reanimar significa no darle el soporte. Entonces eso está mal. Y lo he visto mucho*”. (ID 55)

El equipo de salud valora el principio de autonomía de los pacientes y la subrogancia de la familia por sobre las decisiones tomadas por el médico. Basado en este principio, el paciente tiene derecho a otorgar o denegar su voluntad para someterse a cualquier tratamiento que tenga como efecto prolongar artificialmente su vida, sin perjuicio de mantener las medidas de soporte ordinario. Para el correcto ejercicio del derecho, los profesionales tratantes están obligados a proporcionar información completa y comprensible, en un ambiente de respeto y soporte^50^. Sin embargo, existe cierto enfoque paternalista cuando el paciente es joven, minoritario entre médicos y enfermeras, que predomina sobre la autonomía, expresando afecto, preocupación, protección, compasión y tristeza, con sensación de impotencia, y/o aumento de la ansiedad e incertidumbre. Esto, en ocasiones, fomenta la toma de decisiones por parte del equipo sin considerar la opinión del paciente, o sin informar de manera comprensible acerca de su pronóstico y manejo. Una manera de lidiar con este enfoque de atención paternalista es la educación, ya que mejora la autonomía de los pacientes respecto de la orden de no reanimar, así como la tasa de supervivencia y la calidad de vida^51^.

El Decreto 41 es un reglamento que complementa la Ley 21375 y que regula las condiciones y la forma en que se proporcionan los cuidados paliativos a la persona con enfermedad terminal o grave. Allí, la declaración de una enfermedad terminal o grave permite a la persona suscribir ante las autoridades pertinentes una declaración de voluntad anticipada que contendrá sus decisiones de carácter vital, para el evento en que no pueda expresar su voluntad o preferencias, las que en todo caso, tendrán como límite el no acelerar artificialmente el proceso de muerte. Asimismo, podrá solicitar su alta voluntaria y también podrá ser solicitada por el apoderado que el paciente haya designado o sus parientes. Como requerimiento de esta ley, los establecimientos deben contar con información destinada a la población general sobre los derechos de las personas con enfermedad terminal o grave descritos en la Ley 21375 y la Ley 20584 que regula los derechos y deberes que tienen las personas en relación con las acciones vinculadas a su atención en salud y describe, además, la seguridad en la atención del paciente^50^. Según Motta *et al*.^52^, una atención segura implica que se cumplan las normas y protocolos en lo referente a la seguridad del paciente, la calidad de atención y el derecho a un trato digno entre otros, teniendo en cuenta, además, que otra dimensión relevante es el resguardo de la privacidad, considerando que la muerte es un acto íntimo, privado y personal.

## CONCLUSIÓN

En la comprensión de la limitación del esfuerzo terapéutico se reconoce la disonancia y heterogeneidad entre el concepto y la práctica, buscando dar respuesta a un proceso de morir respetuoso y digno, generando información, para evitar la obstinación terapéutica e incrementar las decisiones al final de la vida de forma oportuna, con fundamentos clínicos y bioéticos, reflejo del ejercicio de la prudencia.

La conceptualización de limitar el esfuerzo terapéutico y la orden de no reanimar aborda cuestiones morales relacionadas con la proporcionalidad de las medidas, prioriza la dignidad y el beneficio del paciente. Sin embargo, se observa falta de consenso y claridad conceptual, lo que puede generar vulneración de derechos del paciente y angustia moral en el equipo de salud. La situación requiere conocimientos claros, directrices precisas respeto a la autonomía de los pacientes, con adecuada información, competencia profesional y capacitación continua.

En cuanto a los cuidados al final de la vida y cuidados paliativos, se observan diversos matices y perspectivas de los temas, no solo por la experiencia personal, sino por la falta de formación en temas bioéticos y legales relacionados con los cuidados al final de la vida.

Se reconoce que la responsabilidad clínica y ética recae en el médico, pero la fuerza de la decisión puede depender de la participación y deliberación de todo el equipo sanitario, el paciente y su familia, generando desafíos éticos en cuanto a la creación de consensos y procesos para entregar información, mantener la confidencialidad, acompañar a las familias, y respetar las voluntades anticipadas de los pacientes.

En cuanto a las limitaciones de este estudio, por falta de recursos, la interpretación de datos no se realizó de forma diferenciada entre técnicos y profesionales; sin embargo, en un segundo estudio sería posible considerar este análisis comparativo. Además, la muestra se seleccionó en el hospital en donde trabaja una de las investigadoras, por ello, sería importante ampliar la segunda parte del estudio, a una mayor cantidad de hospitales.

Por último, es posible adherir a las recomendaciones *Worldwide end-of-life practice for patients in intensive care units* (WELPICUS)^19^, que consensuó prácticas mundiales al final de la vida para las unidades de cuidados intensivos, con la participación de equipos multidisciplinarios. Esta debiera ser una base para proponer capacitación, deliberación, nuevos enfoques, y adaptaciones para establecer las mejores prácticas del cuidado al final de la vida.
