# Supplementary material for: Orden de no reanimar y limitación del esfuerzo terapéutico: desafíos éticos en equipos sanitarios en Chile
Source: Salud Colect. 2024 Jun 5;20:e4821. [Article in Spanish] doi: 10.18294/sc.2024.4821 (PMC11925742; doi:10.18294/sc.2024.4821)
Supplement: Translation

# Translation: Do not resuscitate orders and limitation of therapeutic effort: Ethical challenges in healthcare teams in Chile

**Authors:** Ana López-Ávila, Edith Rivas-Riveros, Maggie Campillay-Campillay

**Affiliations:** 1 Master in Clinical Epidemiology. Clinical nurse, Hospital Regional de Talca, Chile. a.lopez89117@gmail.com; 2 Doctor in Nursing. Director, Master’s Degree in Nursing, Universidad de La Frontera, Temuco, Chile. edith.rivas@ufrontera.cl; 3 Doctor in Nursing. Coordinator, Master of Research in Qualitative Methodologies for Health, Universidad de Atacama, Copiapó, Chile. maggie.campillay@uda.cl

**Keywords:** Do-Not-Resuscitate Order, End-of-Life Care, Bioethics, Health Personnel, Chile

## Abstract

The purpose of this paper is to delve into the ethical aspects experienced by the healthcare team when they receive the directive to limit therapeutic effort or a do-not-resuscitate order. From an interpretative, qualitative paradigm with a content analysis approach, a process based on three phases was conducted: pre-analysis in which categories were identified, the projection of the analysis, and inductive analysis. During 2023, interviews were conducted in the clinical setting of a high-complexity hospital in Chile with 56 members of the healthcare teams from critical and emergency units, from which four categories emerged: a) the risk of violating patients’ rights by using do-not-resuscitate orders and limiting therapeutic effort; b) the gap in the interpretation of the legal framework addressing the care and attention of patients at the end of life or with terminal illnesses by the healthcare team; c) ethical conflicts in end-of-life care; and d) efficient care versus holistic care in patients with terminal illness. There are significant gaps in bioethics training and aspects of a good death in healthcare teams facing the directive to limit therapeutic effort and not resuscitate. It is suggested to train personnel and work on a consensus guide to address the ethical aspects of a good death.

## INTRODUCTION

The various historical and social events that question paternalistic care, as well as the risks and benefits of new health technologies, have contributed to the development of care bioethics. This has challenged the blind faith in medicine, paving the way for a more reflective society that demands greater responsibility from those who practice it^1^. One of the most discussed cases regarding patient rights and the limitation of therapeutic efforts was the request by the parents of Karen Ann Quinlan in the United States in 1976. They opposed keeping their daughter on a respirator to artificially prolong her life while she was in a vegetative state, creating an ethical and legal conflict with the medical team responsible for her treatment. The team was in favor of maintaining respiratory support at all costs, as its withdrawal was considered morally questionable by them^2^. The judge ruled in favor of removing the respirator, and Karen survived with signs of physical deterioration for some time, with the help of enteral feeding and hydration. In response to this case, care ethics committees began to be established at the hospital level to contribute to ethical deliberation, advise teams on ethical conflicts in clinical practice, and avoid therapeutic obstinacy^2^^,^^3^.

Along with the sustained advancement of technology use in medicine and the increased life expectancy of the population, discussions about the right to die with dignity have also emerged, with significant sociocultural nuances. This is mainly because end-of-life decisions are influenced by family affiliation patterns, religious beliefs, and conceptions of death that vary across different regions of the world^4^. An example of this in the Western world is the population’s avoidance of discussing death, which has made it difficult to prepare healthcare professionals to adequately face the stage of anticipatory grief with hospitalized patients suffering from serious illnesses, and to manage the relationship between the dying person and their family. In this context, dying is not only an experience shared by the healthcare team and the patient but also a responsibility shared with the institution, family members, friends, religious figures, and community groups. Follow-up studies on the care of dying patients conducted by Kübler Ross described that the team responsible for these patients’ care primarily focused on physical and medical care. However, dying individuals spent long periods alone, as the team avoided psychosocial and spiritual accompaniment, having not resolved their own perspective on death. Therefore, ongoing training and the humanized formation of critical care teams are essential^5^.

In Europe and later in Latin America, the criteria of the Joint Commission on Accreditation of Health Care Organizations were incorporated into the accreditation of hospital quality, reinforcing the institutionalization of ethical debates through ethics committees. These committees have enabled progress on issues of dignity, autonomy, and end-of-life care for patients admitted to the critical care units of the most complex hospitals^3^.

In Chile, the prolongation of the dying process has been influenced by the limitation of therapeutic effort and the do-not-resuscitate order, concepts addressed in Law 21375 and Law 20584. These provisions have altered the relationship between patients and healthcare teams, favoring the right of individuals to be treated with greater dignity during their therapeutic journey, to access comprehensive care, and to receive relief from the suffering associated with a terminal or serious illness^6^^,^^7^.

The concept of limitation of therapeutic effort has evolved over time, as it is not limited to therapeutic effort alone but includes an adjustment to therapeutic objectives. Therefore, some authors have opted to use the term “adjustment of therapeutic effort”^8^. Conceptually, the limitation of therapeutic effort involves the withdrawal or non-initiation of therapeutic measures because they are deemed useless or futile based on the patient’s medical condition. In this sense, continuing therapeutic measures would only serve to prolong biological life without the possibility of providing functional recovery or a minimal quality of life^9^. Moreover, in terminal phases of the illness, it is a matter of discussion when the focus of treatment has shifted from curative to palliative, thus aiming to alleviate pain and suffering and to maximize patient comfort without prolonging the dying process. This should be done respecting the wishes of the patient and the family and reasonably consistent with the ethical, cultural, and clinical standards of each country^8^^,^^10^. Therefore, the limitation of therapeutic effort seeks a proportional adjustment of treatment, resulting from a deliberation between the patient and the professional, based on a participatory and trustful relationship as an expression of respect for the patient’s personal dignity and humanity ^10^. Practically, it is considered a framework to guide the healthcare team during the care process ^(^^11^.

From the perspective of the bioethical principles that underpin the limitation of therapeutic effort, the following are key: fulfilling the goals of medicine, avoiding unnecessary harm and suffering, allowing a peaceful death, respecting the patient’s values and wishes, maintaining the proportionality of therapies, and seeking rationality in the use of resources based on the principle of justice^10^. Thus, the proportionality of therapeutic effort always refers to a specific clinical situation, which should result from extensive deliberation by the medical team, always leaving open the possibility of reconsideration^12^.

One of the implications of limiting therapeutic effort is the restriction of a patient’s admission to an intensive care unit. Attempts have been made to define criteria for limiting the admission of patients who will not benefit from this decision, considering the need for active monitoring, the reversibility of the process, and the impossibility of performing the necessary treatment outside of this unit^13^. Thus, decision-making in this regard responds to the patient’s conditions not only physically but also their consciousness to participate, as much as possible, in these decisions. Therefore, the criteria to consider must be both technical and ethical. These decisions are not exempt from moral conflicts, especially since they must take into account that it is most likely that the decisions will be made by family members of patients at risk of death^14^. Technical criteria are related to the probability of survival and the benefits of using advanced technology to maintain patients with less suffering and better quality of life. In this sense, international experience has linked a “good death”^15^ with the use of the order to limit therapeutic effort within the first 48 hours of the patient’s admission. Therefore, the process must be clearly recorded and documented in the medical record. This includes justifying the indication and documenting whether the family is aware of such a decision and if they agree with it or not. Additionally, there is a medical obligation and moral duty not to abandon the patient during the dying process, ensuring the necessary measures for well-being, care, analgesia, and sedation, ensuring the absence of physical or psychological pain^15^.

On the other hand, the “do-not-resuscitate” order is an advance directive in which a physician instructs the healthcare team not to initiate cardiopulmonary resuscitation in a patient if their heart stops or if they cease to breathe^16^,^17^.

It is a topic related to human dignity, patient rights, the dying process, and it requires interpersonal communication skills with the patient, team, and family. The limitation of therapeutic effort is justified from a perspective of justice (avoiding futility), non-maleficence (avoiding therapeutic obstinacy), beneficence (respecting the dignity of the individual), and patient autonomy (respecting their own or their family’s will)^18^. In the decision not to resuscitate, the ethical principles of therapeutic proportionality, the exercise of responsible freedom, and the inviolability of human life acquire special preeminence, given that such determination results in the certain, unavoidable, and immediate death of the person affected by a cardiopulmonary arrest^12^. In this scenario, the patient’s advance directive must be respected, although it is not always possible for all patients to want to discuss this possibility, and the same may occur with the family. Likewise, physicians consider discussing this issue with the patient and family challenging, as it may alter the physician-patient relationship and diminish hope for the family^16^. Another sensitive aspect is the privacy with which this issue is handled, considering that the codes adopted to communicate the order to limit therapeutic effort to healthcare personnel should not be decipherable to other patients. In this regard, health personnel education becomes essential to address this situation, respecting the confidentiality and dignity of the dying process of every patient ^19^.

Therefore, the aim of this study is to delve into, from a qualitative approach, the bioethical aspects experienced by the healthcare team when they receive the indication to limit therapeutic effort and the do-not-resuscitate order. It is hoped that this will contribute to the informed discussion among healthcare teams, focusing on patient-centered care and the patient as a subject of rights.

## METHOD

An exploratory study was conducted from an interpretative paradigm and qualitative approach. The sample was intentional, consisting of healthcare professionals and technicians (physicians, nurses, physiotherapists, and nursing technicians) working in the critical care units, internal medicine hospitalized units, and emergency department of a high-complexity hospital in the southern region of Chile. These units are characterized by providing care to patients at life-threatening risk, so it is common for these teams to use cardiac monitoring technology, respiratory assistance, catheters, and other advanced medical technologies, and to face end-of-life decisions. The hospital management was consulted for the number of staff working in these units, and voluntary invitations were sent to service heads so that at least 20 people per service could participate. They facilitated contact with their teams, receiving a positive response from 20 informants per unit. However, in the emergency department, four of the voluntary staff subsequently declined to participate, resulting in a total sample of 56 subjects. The interview period extended over six months during the year 2023.

Inclusion criteria considered members of teams engaged in direct patient care activities, with at least one year of experience in one of the services of interest. The data collection technique was through semi-structured interviews, using a script with four guiding questions, including: “What do you understand by limitation of therapeutic effort, do-not-resuscitate order, and their relationship with end-of-life care and the concept of a good death for patients?” This allowed guiding the course of the interview to deepen the study’s objectives and give greater emphasis to emerging themes. Data collection began once the evaluation and approval of the Scientific Ethics Committee of the University of La Frontera, recorded in minutes No. 67/23, along with written authorization from the management of the institution where the research was conducted, were obtained.

The interviews were conducted in a room within the same hospital, and COVID-19 prevention protocols were observed. The average duration of the interviews was 67 minutes, with a range of 45 to 90 minutes. The interviews were consented with physical recording, and a digital recorder was used for voice recording. Additionally, field notes were taken to record any observations that would aid in the subsequent interpretation of the data. Transcription was done faithfully, and a storage file was created with anonymized interviews and the assignment of an informant code “ID” accompanied by a sequential number. Finally, manual analysis was opted for due to the lack of access to software with a valid license.

The credibility criterion considered triangulation by cross-referencing data between the principal researcher and secondary researchers, who contributed to interpretation, had experience in qualitative studies, and provided feedback for greater reflexivity based on commitment and interest in the subject. Saturation was achieved, as once the emerging categories were established, it was possible to identify similarities and scarce new information among the informants. Similarly, while the results are from a single hospital, the similar characteristics in the training of healthcare professionals in our country may possibly yield transferable results to other critical care teams in other hospitals in Chile^20^.

This is a qualitative content analysis, following the qualitative approach of Bardin^21^. It was conducted as follows: a) a pre-analysis phase where categories were identified, b) a second phase of analysis projection, and c) a third phase where data were analyzed logically and inductively to meet the study objectives. The pre-analysis was independently performed by the principal investigator and collaborators. All possible categories that progressively emerged as a result of the different rounds of data reading were listed. In the second phase, thematic categories considered substantive were identified, both due to the content trend and the importance associated with the objectives. Finally, in the third phase, the researchers chose to prioritize certain categories, as many were properties or characteristics of others. In summary, the emerging thematic categories were: a) risk of violating patients’ rights when using the “do-not-resuscitate” order and “limitation of therapeutic effort”; b) gap in the interpretation of the legal framework; c) ethical conflicts in end-of-life care; and d) efficient care or holistic care in patients with terminal illness.

## RESULTS AND ANALYSIS

### Sample characterization

A total of 56 healthcare professionals and technicians (physicians, nurses, physiotherapists, and nursing technicians) from critical patient, medicine, and emergency services were interviewed. They had varied years of experience, with an average age of 37 years (Table 1).

**Table 1:** Sample characterization. Talca, Chile, 2023.

Characteristics of the informants	Number of informants	Average age	Unit			Average years of experience
			Critical patient unit	Medicine	Emergency	
Technicians	20	38	6	9	5	10.5
Nurses	18	35	7	5	6	9.0
Physiotherapists	8	34	4	2	2	9.8
Physicians	10	41	3	4	3	14.6
Totals	56	-	20	20	16	-

Source: Own elaboration.

### Risk of violating patients’ rights when using the “do-not-resuscitate” order and limitation of therapeutic effort

Informants mention that there is a common use of these orders in the studied units; however, there are variations in how the team interprets and implements them in clinical practice. In this sense, most mention that the do-not-resuscitate order is a literal order, meaning that patients with this indication should not receive cardiopulmonary resuscitation measures due to their advanced overall compromised state.

“In the event that the patient goes into cardiopulmonary arrest, they will not receive cardiopulmonary resuscitation, they will not be intubated if needed, or anything like that. No resuscitation. This does not mean that they will not continue to receive their treatment as appropriate, their nutrition and everything, until their last breath.” (ID 34)

On the other hand, when faced with the indication of therapeutic effort limitation, they mention that while they understand the medical context that justifies it, there are difficulties in deciding how to act appropriately with the patient:

“I thought it was the same, when they limit it and in the end, they don’t resuscitate. What the difference is, I couldn’t say.” (ID 4)

“When we are instructed to limit therapeutic efforts, it is mostly about limiting invasive therapies or procedures when the patient’s prognosis is poor and it really reaches a ceiling. For example, there may be supportive measures, but we are no longer going to innovate with other things. So there is a limitation in terms of activity. Well, for us here, sometimes it creates a bit of a problem, I think it’s interpretative. Sometimes, the person indicating it as well, what do we really want in the end? Do we want to maintain the patient, do we want to remove some supportive measures? And I think there are interpretation problems there because sometimes one shift limits it, and then suddenly the other doctor indicates other things, so maybe there is a need to come to an agreement on these things.” (ID 37)

At a global level, the literature mentions that the application of such terms generates practical and ethical complications because there are a large number of alternative semantic expressions^22^. Furthermore, these orders are commonly associated with the acronyms DNR (do-not-resuscitate) or TEL (therapeutic effort limitation), which are widely used alongside other acronyms among healthcare personnel and can lead to misinterpretations, thus implying the possibility of errors in the execution of the instructions^23^, especially among less experienced staff. Another relevant aspect is that therapeutic decisions related to prognosis and patient management are the responsibility of physicians, and in the case of nurses or other healthcare professionals involved in carrying out the do-not-resuscitate and therapeutic effort limitation orders, they do not always participate in deliberations regarding the most advisable and beneficial aspects for the patient, which may affect mental health problems when executing the order. However, there is an increasing trend towards consulting the entire team, which undoubtedly contributes to enabling the entire team to better cope with these instructions^19^^,^^24^.

In this regard, the main obstacle for healthcare teams is to correctly interpret the instructions, which can influence actions to ensure that patients die with dignity and comfort. Beyond the standardization of acronyms, knowledge about end-of-life care, and becoming familiar with the legal framework of each country are fundamental to reduce the risk of error and prevent the violation of patients’ rights^19^. According to the American Nurses Association^25^, supporting end-of-life patient decisions should be done consistently with their preferences and values, and it is related to a proper understanding of the medical terminology used. Therefore, the use of acronyms, abbreviations, and initials with the patient and family should be discouraged, as they often lead to communication problems.

When asked about the benefits of therapeutic effort limitation for end-of-life patients, the interviewed individuals associate it with improved quality of life, comfort, death with dignity, and not abandoning the patient. These ideas underpin the benefits for patients of using therapeutic effort limitation in order to not prolong persistent physical suffering that serious and terminal illnesses can cause these individuals^6^. Additionally, while the use of these instructions in critical patients causes moral distress and strong emotions that are difficult to manage for professional teams^26^, participating in care planning, along with improving the end-of-life condition of these patients, brings peace of mind to clinical teams under the idea that they have acted doing everything possible for the patient:

“If the patient is conscious, they are afraid of feeling pain, they are afraid of feeling suffocated. So, for me, regarding symptoms, it means that they don’t have to feel that. And regarding the emotional part, it is also about being accompanied, not going through this process alone. Also, if the patient has the desire to see people they haven’t seen in a long time, fulfilling those last wishes that are feasible, that is what having a good death means to me.” (ID 16)

“If one does the utmost to ensure they have a good death, one leaves more peacefully. That is a lesson, because sometimes, due to certain circumstances, it is not achieved. Whenever one passes through afterwards, one thinks, ‘I could have done this, I could have done that,’ it gives you more confidence for when there is a plan, that gives you an experience in achieving a good death for patients.” (ID 6)

When asked about end-of-life care, the informants associate it with holistic care and support, emphasizing the importance of caring for all human dimensions - physical, psychological, and social - at this stage:

“*End-of-life care is about providing comfort to the patient. Especially in terminal illnesses, pain management, family support, psychiatric and psychological care, especially for the relatives.” (ID 9)*

Regarding palliative care, this is associated with providing care for terminally ill or cancer patients. At this point, special mention is made of pain management, comfort, and companionship, very similar to end-of-life care. However, the informants identify patients who, due to their illness, are experiencing a more painful agony:

“Palliative care is, for example, for oncology patients who are already very complicated with their illness, their condition. It’s about providing them with the necessary medications so that they don’t have much pain, giving them their medications, like good end-of-life care, that’s what I understand as palliative care. It’s about the patient reaching their last day in a way, without pain, and peacefully.” (ID 4)

The care of patients with painful terminal illnesses, such as cancer, deteriorates the patient’s quality of life and affects their family environment. In these cases, pain refers not only to physical perception but also to suffering associated with psychological distress, loss of social activities, and family grief. The study conducted by Parra et al.^27^ in a pediatric oncology service described the significant role played by the family in pain management as a multifactorial process. Along with the constant accompaniment of the family to children, authorization from them is required for analgesic treatment to prevent physical suffering due to their heteronomous condition. Other studies have described the importance of the personal element, as each human being experiences their pain process differently. At this point, the informants use the concept of “patient comfort” to refer to any activity that promotes comfort, pain control, and meaningful patient and family support. The review by Zamán et al.^28^ on dying well is consistent with the findings of this study, emphasizing that the terminal patient should die in their preferred place, with pain relief and without psychological distress, with emotional support from loved ones and autonomous decision-making, when possible, and avoiding prolonging life by limiting futile interventions.

Pain is one of the most mysterious human experiences; the physical suffering of terminal patients is also associated with psychological and spiritual suffering, which is experienced personally and whose meaning is a matter of ongoing debate in society. Some groups find spiritual meaning in suffering, which strengthens human dignity, while for others, suffering is senseless and detracts from human life’s dignity^29^. What is clear is that suffering is associated with the condition of sentient beings, exposes our vulnerability, and disrupts the well-being of the person experiencing it^30^. Based on this, the healthcare team must understand what meaning and significance the terminal patient has given to their own pain, how much they are willing to tolerate, and at what point they will require medication support to cope with it.

For the interviewees, both end-of-life care and palliative care involve alleviating the terminal patient’s pain and suffering. This aligns with the literature emphasizing the importance of healthcare teams advocating for a dignified death, providing pain relief, and adequate comfort conditions. Additionally, the need for good communication is emphasized, especially with family, friends, and other significant figures, to provide psychological and spiritual support when the patient requires it^31^.

### Gap in the Interpretation of the Legal Framework

Interviewees mention that the legal framework in Chile, which regulates end-of-life care and terminal illnesses, is insufficient to have adequate care protocols, while other team members are unaware of the legal framework regulating these cases. Additionally, coercive aspects of the law are mentioned, as adequate attention is not given to this group of patients.

In this sense, there are knowledge gaps regarding the legal aspects regulating these issues, a matter that requires urgent attention as it limits professional action and jeopardizes the exercise of patients’ rights.

“*I think that, firstly, this limitation of therapeutic effort and the order of non-resuscitation crosses a boundary between the medical and the legal, because when one strictly observes how the limitation of therapeutic effort is implemented, each country must have legislation on the matter, and Chile does not have it. So everything is left to the discretion of the team caring for the patient. So I think we should unify criteria, create a ministerial protocol that will be applied to have a national uniformity of criteria and there would be no doubt about this.” (ID 53)*

This knowledge gap regarding the legal framework regulating end-of-life care within the healthcare team is complex. The existing sanitary normative framework is the result of a social and democratic debate in which healthcare professionals must participate and stay informed, as they play fundamental roles as providers and caretakers of patients^32^^,^^33^. Furthermore, these laws are operationalized through technical guidelines, which guide the decisions and actions taken by the team. For example, Law 21375 enshrines palliative care and the rights of individuals suffering from terminal or severe illnesses, shaping the Universal Palliative Care Technical Guidance^34^ These guidelines should be incorporated into the lex artis, which corresponds to the set of best practices based on scientific evidence that should guide professionals and healthcare establishments at a national level^33^.

Other studies conducted in Chile have also highlighted gaps in bioethical knowledge. For instance, Reyes et al.^35^ found that 76.6% of specialist physicians in a hospital had a deficit in knowledge regarding informed consent, thereby compromising patient autonomy. Additionally, Strickler et al.’s^36^ study on bioethical knowledge in emergency units describes difficulties in applying bioethical knowledge in clinical practice among physicians and nurses.

According to Caro^37^, “assuming end-of-life care as a public policy grounded in bioethical principles and current regulations should be: appropriate, available, accessible, educational, and implemented in the healthcare system”^37^. This poses significant challenges for decision-makers and those responsible for healthcare administration to advance continuous training of healthcare teams in ethics and legal aspects associated with healthcare. In Chile, there already exists an important regulatory framework that addresses end-of-life care, which is also included in the basket of services of the Explicit Health Guarantees (GES). This set of norms began in 2012 with the approval of Law 20584, which regulates the rights and duties of individuals concerning actions related to healthcare. Nationally, this marks a significant advancement in the therapeutic relationship between the physician and patient by introducing the right to autonomy in decisions related to their health-disease process, replacing the principle of beneficence that determined the paternalistic therapeutic relationship for a long time^33^. Subsequently, Law 21375 was enacted, which enshrines palliative care and the rights of individuals suffering from terminal or serious illnesses, aiming to provide greater dignity to the trajectory of suffering from a critical and/or terminal illness in individuals^6^.

### Ethical conflicts in end-of-life care

The main ethical conflicts identified in the study relate to the patient’s age and the therapeutic effort to sustain life: being elderly is considered a criterion that influences the decision to limit therapeutic effort.

#### The patient’s age and the therapeutic effort to sustain life.

The interviewed individuals acknowledge that age is a factor present in the healthcare team’s reflection when debating whether to limit therapeutic efforts in a patient. The literature on this topic mentions that in intensive care units, age and the quality of life that can be offered to the patient after a critical period, along with their chances of survival, are relevant elements for deciding to limit therapeutic efforts. This generates discussion, as studies conducted in intensive care units globally mention that age is an independent factor of poor prognosis^38^. Maintaining or withdrawing end-of-life care interventions depends primarily on the patient’s medical condition and the high probability of death, lack of response to therapy, severity, and poor outcome in terms of quality of life, and not on age^39^. The literature review conducted by Araújo et al.^40^ describes that older people mostly receive unfair treatment, as they are denied access to diagnostic tests and treatments by the healthcare team, which is solely associated with their advanced age. They also report receiving inadequate treatment and violence from the team, who use inappropriate language and transfer the responsibility of their care to their families, even though their mental state allows them to maintain autonomy. This demonstrates that ageism is strongly institutionalized and affects decisions and opportunities to maintain functionality and a better quality of life for older people. This unfair treatment is a form of structural ageism that leads to poor living conditions for older people, reaffirms discrimination by younger populations, and reduces opportunities to remain active until their autonomy is limited^41^.

“*There are patients who are elderly, patients with multiple comorbidities, whose quality of life may have already deteriorated, and it is easier to face that they are reaching the end of life due to the natural course of their multiple diseases, where it can be faced a bit, the outcome is more expected.” (ID 48)*

“A young patient is more difficult to limit, not to resuscitate, you understand? Whereas a patient, I don’t know, because sometimes you encounter patients who are 100 years old, it’s like, it’s the law of life, I mean, no, no, let’s not do, what’s it called? Oh, I forgot the term. When you do more than you should, medical overtreatment.” (ID 57)

#### Order to limit therapeutic effort and the dilemma of feeling that this action causes harm to the patient

The informants mention that once life-sustaining measures are withdrawn, it becomes essential to maintain basic care for the comfort and well-being of the patient. However, they hint that they feel uncomfortable carrying out this order. This is consistent with the study conducted by Velarde et al.^42^, where nurses who executed the order to limit therapeutic effort felt that they were not only withholding treatment from the patient but also contributing to their death. The interdependent relationship that nurses develop with their patients fosters deep emotional bonds that can lead to negative emotions in the face of end-of-life actions or limit the ability to psychologically cope with the death of these patients. According to Sprung et al.^19^, professionals should receive adequate training to achieve a certain level of competence in caring for terminally ill individuals, becoming experts in decision-making, ethical and practical aspects, while respecting the patient’s viewpoint according to their culture and beliefs.

“*A patient cannot die because you didn’t aspirate them or because a tracheostomy tube got blocked because you didn’t aspirate it. So that’s not correct. We need to be vigilant about changes in position, for patients who are bedridden, the idea is not to let them deteriorate further and add another pain or require plastic surgery. It doesn’t make sense to keep deteriorating the patient and spending resources. Essentially, the goal is for the patient to maintain all possible minimal and quality care.” (ID 41)*

“But there are some ethical elements that concern me, for example, the permeability of the airway. The patient may have a do-not-resuscitate order, but I cannot allow the direct cause of their death to be asphyxiation, for example. I would probably be providing palliative care at that moment.” (ID 34)

#### Efficient care, or holistic care in patients with terminal illness

Some of the informants expressed feelings of frustration regarding the investment of time, resources, and costs associated with the care of a patient who later receives an order to limit therapeutic efforts and dies.

“*The people who work here feel that investment of time could be lost. It could be, I don’t know, that accompanying or being there has no value or that it will be a waste of time compared to what we could do with other people.” (ID 32)*

“*It’s quite frustrating sometimes that one day we see the patient with everything, we culture them, intubate them, give them last-line antibiotics, and then days later they come and take everything out, and you’ve lost all the work you did the day before, because the other doctor said, I mean, it’s not limited, don’t do anything to them, and they make you work extra.” (ID 41)*

“*No exams, it’s a resource that is wasted. I’ve never really thought deeply about it because it’s not my decision. But yes, I would remove everything, everything.” (ID 24)*

Studies on critically ill patients are categorical in establishing that the costs associated with care are high and depend on the type of medical technology used, supplies, and hours of personnel dedicated to the patient^43^. Therefore, questioning the use of resources in a patient who later dies often responds to the principle of justice, as the goal is to benefit the greatest number of patients. From this perspective, taking futile actions to sustain the life of a patient with a poor prognosis is not only a misallocation of resources but also constitutes therapeutic obstinacy and prolongs unnecessary suffering. The dilemma in this case is being able to know when to use resources and when they no longer contribute to the patient’s well-being, as all lives are important and require fair and dignified treatment^44^.

On the other hand, efficiency from an ethical perspective is an instrumental value because, according to Gracia Guillén^45^, it appeals to economic rationality concerned with the cost/benefit of actions, a characteristic of Western civilization that relies on material and utilitarian considerations. Therefore, feeling frustration and mentioning that “resources were lost” when caring for a person who later dies would correspond to a form of “instrumentalized care,” probably unconscious and learned, since individuals do not have a price but dignity. In this sense, Campbell^46^ mentions that dying well from a cultural and holistic perspective should consider four aspects: the place of death, companionship, the cause of death, and the way of facing death. Therefore, concern about the use of resources should be addressed in the context of ensuring patients die well.

The Law 21375 establishes palliative care and the rights of individuals suffering from terminal or severe illnesses, and in Article 4 it states, “the protection of the dignity and autonomy of individuals suffering from a terminal or severe illness always entails respecting their life and considering death as part of the life cycle”^6^. Regarding this, literature on the professional attitude towards a patient’s death is scarce. The study by Morales et al.^47^ described some nurses’ attitudes of indifference towards patients’ deaths, so as not to be emotionally affected. The authors related this to “accepting one’s own death, caring with much more freedom, which can translate into assuming responsibilities for personal and professional growth, and allows for a greater meaning of life”^47^. However, this practice of showing indifference towards death may also reveal difficulty in facing the dying process, gaps in training, and a risk of conveying wrong messages to students who are often impacted when they have their first experiences with death^48^. On the other hand, death is a vital experience that, according to Sprung et al.^19^, requires patient and family-centered care, not only to focus on alleviating symptoms of the illness, preventing or relieving physical suffering, but also to treat people with dignity and address their needs holistically, providing physical, psychological, social, and spiritual care according to the preferences of the patient or family.

The holistic approach to care considers the interaction between the patient and their family as relevant for maintaining emotional bonds and socialization until the end of life, avoiding social isolation, and also promotes psychological and spiritual support^49^.

#### The advance directive of the patient to limit therapeutic efforts or the decision of others?

At this point, the healthcare team values the principle of patient autonomy, with family substitution taking precedence over decisions made by the physician:

“*That the person’s right is done, to know that the health team will not do anything if something happens to them, perhaps it is a right of that person, right? Perhaps that person has the right to know their condition of not resuscitating or their condition of therapeutic limitation. Perhaps that person should know.” (ID 34)*

“*I believe that as a team, we have to know and consider the perspective of the family, consider the perspective of the patient, and not make the decision for them ourselves.” (ID 40)*

“*The do-not-resuscitate order is an indication based on the patient’s clinical condition, but sometimes it’s also defined by the patient themselves and stated in the medical record.” (ID 53)*

“*I believe that the family should also be involved, or what the patient wants should be considered because there are patients who are very alert, who are very oriented in time and space, and who are aware of their situation and are able to make a decision about what they want, but here the clinicians don’t have a clear concept, and therefore the fact of a do-not-resuscitate order means not giving them support. So that’s wrong. And I’ve seen it a lot.” (ID 55)*

The healthcare team values the principle of patient autonomy and family substitution over decisions made by the physician. Based on this principle, the patient has the right to grant or deny their will to undergo any treatment that artificially prolongs their life, without prejudice to maintaining ordinary supportive measures. For the correct exercise of this right, treating professionals are obligated to provide complete and understandable information in an environment of respect and support^50^. However, there is a certain paternalistic approach when the patient is young, which is a minority among doctors and nurses, that predominates over autonomy, expressing affection, concern, protection, compassion, and sadness, with feelings of powerlessness, and/or increased anxiety and uncertainty. This, at times, encourages decision-making by the team without considering the patient’s opinion, or without comprehensively informing them about their prognosis and management. One way to deal with this paternalistic approach to care is education, as it improves patient autonomy regarding the “do not resuscitate” order, as well as survival rates and quality of life^51^.

Decree 41 is a regulation that complements Law 21375 and regulates the conditions and manner in which palliative care is provided to individuals with terminal or serious illnesses. There, the declaration of a terminal or serious illness allows the individual to subscribe to an advance directive before the relevant authorities, which will contain their vital decisions for the event that they cannot express their will or preferences, which in any case, will have as a limit not to artificially accelerate the dying process. Likewise, they may request voluntary discharge, which can also be requested by the proxy appointed by the patient or their relatives. As a requirement of this law, establishments must provide information intended for the general population about the rights of individuals with terminal or serious illnesses described in Law 21375 and Law 20584, which regulates the rights and duties of individuals in relation to actions linked to their healthcare and also describes patient care safety^50^. According to Motta et al.^52^, safe care implies compliance with standards and protocols regarding patient safety, quality of care, and the right to dignified treatment, among others, considering also that another relevant dimension is safeguarding privacy, considering that death is an intimate, private, and personal act.

## CONCLUSION

In understanding the limitation of therapeutic effort, there is recognition of dissonance and heterogeneity between the concept and practice, aiming to provide a response to a dying process that is respectful and dignified, generating information to avoid therapeutic obstinacy and increase end-of-life decisions in a timely manner, with clinical and bioethical foundations, reflecting the exercise of prudence.

The conceptualization of limiting therapeutic effort and the do-not-resuscitate order addresses moral issues related to the proportionality of measures, prioritizing the dignity and benefit of the patient. However, there is a lack of consensus and conceptual clarity, which can lead to the violation of patient rights and moral distress among healthcare teams. The situation calls for clear understanding, precise guidelines regarding patient autonomy, with adequate information, professional competence, and ongoing training.

Regarding end-of-life care and palliative care, various nuances and perspectives on the topics are observed, not only due to personal experience but also due to a lack of training in bioethical and legal issues related to end-of-life care.

It is recognized that clinical and ethical responsibility falls on the physician, but the strength of the decision may depend on the participation and deliberation of the entire healthcare team, the patient, and their family, generating ethical challenges regarding the creation of consensus and processes for providing information, maintaining confidentiality, supporting families, and respecting patients’ advance directives.

Regarding the limitations of this study, due to resource constraints, data interpretation was not differentiated between technicians and professionals; however, in a subsequent study, it would be possible to consider this comparative analysis. Additionally, the sample was selected from the hospital where one of the researchers works; therefore, it would be important to expand the second part of the study to a larger number of hospitals.

Finally, it is possible to adhere to the recommendations of the *Worldwide End-of-Life Practice for Patients in Intensive Care Units* (WELPICUS)^19^, which reached consensus on global end-of-life practices for intensive care units, with the participation of multidisciplinary teams. This should serve as a basis for proposing training, deliberation, new approaches, and adaptations to establish the best end-of-life care practices.
